# Related male *Drosophila melanogaster* reared together as larvae fight less and sire longer lived daughters

**DOI:** 10.1002/ece3.1549

**Published:** 2015-06-24

**Authors:** Pau Carazo, Jennifer C Perry, Fern Johnson, Tommaso Pizzari, Stuart Wigby

**Affiliations:** 1 Edward Grey Institute, Department of Zoology, University of OxfordOxford, UK; 2 Cavanilles Institute of Biodiversity and Evolutionary Biology, University of ValenciaValencia, Spain; 3 Jesus College, University of OxfordOxford, UK

**Keywords:** *Drosophila*, familiarity, kin recognition, kin selection, maternal effects, sexual conflict, transgenerational effects

## Abstract

Competition over access to reproductive opportunities can lead males to harm females. However, recent work has shown that, in *Drosophila melanogaster*, male competition and male harm of females are both reduced under conditions simulating male-specific population viscosity (i.e., in groups where males are related and reared with each other as larvae). Here, we seek to replicate these findings and investigate whether male population viscosity can have repercussions for the fitness of offspring in the next generation. We show that groups of unrelated-unfamiliar (i.e., unrelated individuals raised apart) males fight more intensely than groups of related-familiar males (i.e., full siblings raised together as larvae), supporting previous findings, and that exposure to a female is required to trigger these differential patterns of male–male competition. Importantly, we show that differences in male–male competition can be associated with transgenerational effects: the daughters of females exposed to unrelated-unfamiliar males suffered higher mortality than the daughters of females exposed to related-familiar males. Collectively, these results suggest that population structure (i.e., variation in the relatedness and/or larval familiarity of local male groups) can modulate male–male competition with important transgenerational consequences.

## Introduction

Sexual selection often favors male adaptations that are harmful to the females over which males compete (Andersson 1994; Arnqvist and Rowe 2005; Parker 2006). Male-derived female harm can have pronounced repercussions for populations as a whole, slowing population growth rate and even leading to local extinctions in a process akin to a “tragedy of the commons” (Le Galliard et al. 2005; Rankin and Kokko 2006; Rankin et al. 2011). Much less appreciated, however, is the extent to which male-induced female harm influences fitness traits in the resulting offspring. This can be important because estimating fitness consequences exclusively in terms of individual payoffs within a generation can be misleading in the presence of parental effects with transgenerational consequences (Priest et al. 2008a).

In principle, variation in the genetic relatedness and social familiarity of local competitors, dictated by population structure, can modulate male–male competition and male harm of females through both direct and indirect fitness effects (Johnstone and Cant 2008; Rankin 2011; Wild et al. 2011; Pizzari and Gardner 2012; Pizzari et al. 2015). Consistent with this, a study of the fruit fly *Drosophila melanogaster* found that groups of males that were full sibs and reared together as larvae (i.e., related-familiar) fought less intensely than did groups of males that were unrelated and reared apart as larvae (i.e., unrelated-unfamiliar; Carazo et al. 2014). Moreover, females exposed to groups of related-familiar males exhibited longer reproductive life span and slower reproductive aging and thus had higher lifetime reproductive success than females maintained with groups of unrelated-unfamiliar males (Carazo et al. 2014), a pattern recently replicated by an independent study (Hollis et al. 2015).

If male population structure influences female fitness, it might also regulate offspring performance through parental effects. One mechanism that could potentially generate this pattern is that male harm of females associated with variation in male relatedness and familiarity might directly influence maternal investment. This possibility is particularly relevant because recent *D. melanogaster* studies have indicated that intersexual interactions can result in complex transgenerational effects (Priest et al. 2008a,b; Dowling et al. 2014). On the one hand, Priest et al. (2008a,b) reported that females exposed to a high mating frequency suffered from shorter life spans and lower lifetime reproductive success than females subjected to medium or low mating frequencies, but produced daughters with higher early fecundity than the daughters of females exposed to medium or low mating frequencies. In contrast, Dowling et al. (2014) recently found that increased intersexual precopulatory interactions can generate survival and aging costs in offspring. Another potential mechanism generating transgenerational effects is differential sib competition. Average offspring relatedness will be higher if a female’s mates are related to one another (e.g., if populations are structured), and this may relax sib competition (Shaanker et al. 1988; Godfray 1995; Mock and Parker 1998). To gain a comprehensive appreciation of the evolutionary implications of male-induced female harm in *D. melanogaster*, it is therefore necessary to measure the effect that male population structure has not only on the females with which males directly interact, but also on their offspring.

**Figure 1:**
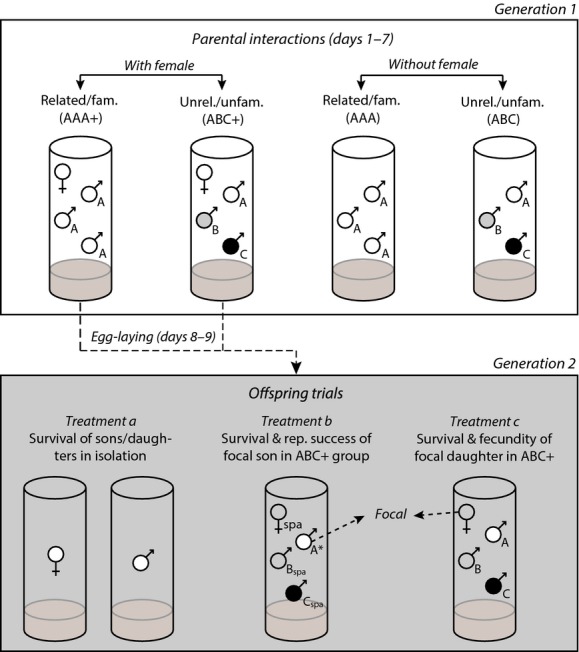
Scheme of experimental design. In generation 1, individual females were exposed to three males which were unrelated to the female and either full-sib brothers raised together (related-familiar, AAA+ treatment) or unrelated to each other and raised apart (unrelated-unfamiliar, ABC+). The offspring produced by these females (generation 2) were exposed to separate trials to measure: the survival of individual sons and daughters in isolation, the survival and reproductive success of daughters exposed to triplets of unrelated-unfamiliar males to each other and to the female (ABC+), and the survival and reproductive success of sons exposed to a female and two other unrelated-unfamiliar males (ABC+).

We had two aims in this study. First, we sought to replicate the finding that male population structure (i.e., local relatedness and larval social familiarity) in *D. melanogaster* relaxes male–male competition for females, as reported by our previous study (Carazo et al. 2014). Second, we tested whether the effects of within-group male relatedness and larval familiarity extend to the next generation. To do so, we first exposed each parental female (generation 1) to groups of three males (unrelated to the female) that were either related and familiar to each other (i.e., full sibs, raised together as larvae), or unrelated and unfamiliar to each other (i.e., raised in their own families as larvae and then mixed between families after emergence). We thus manipulated larval familiarity (while controlling for adult familiarity) and relatedness as proximal cues of population structure. We then tested for the influence of these two aspects of population structure on the intensity of male–male competition by measuring male–male aggression and courtship behavior over 1 week. We also compared male–male aggression observed under these conditions to male aggression measured in the absence of females, to test whether male aggression is associated with competition for mates. We then isolated the parental females, collected their offspring, and measured a suite of fitness-related traits (life span, early female productivity, and male mating success) in the offspring (generation 2; Fig.1), to test whether male relatedness and larval familiarity are associated with transgenerational effects.

## Methods

### Stock cultures

We used a laboratory-adapted, wild-type Dahomey stock of *D. melanogaster*, maintained since 1970 in large outbred populations with overlapping generations (Partridge and Farquhar 1983). The recessive *sparkling poliert* (*spa*), mutation was backcrossed into the wild-type Dahomey population for at least five generations. *spa* flies posses a rough eye phenotype that allows us to visually differentiate between the offspring of *spa* parents and those of wild-type parents. All flies were maintained in a 25°C, nonhumidified room, with a 12:12 h Light:Dark cycle. Experiments were carried out in 36-mL plastic vials containing Lewis medium (Lewis 1960) with excess live yeast grains. Virgin flies were collected within 8 h of eclosion using ice anesthesia.

### Generating families and parental females (generation 1)

To grow experimental flies, we collected Dahomey eggs from population cages on grape-agar filled Petri dishes, smeared with live yeast paste. The eggs were placed at a standardized density of 10 *μ*L (∼180 eggs) per 75 mL bottle containing ∼45 mL fly food using the protocol described in Clancy and Kennington (2001). Virgin females were collected from these bottles within 8 h of eclosion and aged in groups of 15 in vials for 48–72 h before the trials. Females were thus unrelated to one another and to the experimental males.

Experimental males were raised in families, among full sibs, until emergence as adults. To generate these families, we first placed pairs of 7 day old virgin Dahomey males and females (reared as above) in a vial with live yeast for 36 h, allowing the female plenty of time to oviposit, after which time the parental pair was discarded. The offspring were reared in these family vials (i.e., in the vial in which they had been laid as eggs) until emergence as adults, at which time they were introduced in their experimental vial (see below). Families were randomly assigned to one of 50 “sets” of families. Each set was composed of three families that were randomly assigned a role as an A, B, or C family (i.e., each set comprised one A, one B, and one C family). Within 6 h of eclosion, male flies from families A, B, and C within each set were used to construct two replicate “related-familiar” vials containing three full-sib males from family A, familiar to each other, and two replicate “unrelated-unfamiliar” vials containing three unrelated males, one from each family, and unfamiliar with each other. These males were left together in vials for 48 h before the beginning of behavioral observations (see below) to ensure that adult social familiarity was standardized across treatments (as opposed to larval social familiarity; Carazo et al. 2014).

### Behavioral assays in parental flies (generation 1)

Virgin Dahomey females were individually placed into one of the two “related-familiar” and into one of the two “unrelated-unfamiliar” vials in each set to produce the following four parental treatments within each set: (1) AAA+, containing three full-sib familiar males and one unrelated virgin female; (2) ABC+, containing three unrelated-unfamiliar males and one unrelated virgin female; (3) a control AAA, containing three full-sib familiar males (no females); and (4) a control ABC, containing three unrelated-unfamiliar males (no females). For each treatment, the sample size was 50. To test whether male relatedness/familiarity affects levels of male–male competition, we conducted behavioral observations during the first 5 day of interactions. Observations were conducted for 2 h following lights on each day, during which time vials were scanned ca. every 20 min by a single observer who was blind to the male relatedness/familiarity treatment of each vial. We recorded male–male aggression rates (i.e., average number of charging or boxing events per scan, as previously described Dierick 2007; Chen et al. 2002), courtship rate (the proportion of scans in which courtship events were observed; Bastock and Manning 1955), and courtship intensity (number of courting males when courting was observed; Carazo et al. 2014). Triplets and foursomes were transferred to fresh vials on day 4, and the interaction period ended on day 7 (see below).

### Transgenerational effects on offspring (generation 2)

We tested whether male relatedness/familiarity was associated with differential transgenerational effects on offspring in the following way. We allowed the experimental foursome treatments (i.e., AAA+ and ABC+) of generation 1 to interact for a total of 7 days (i.e., the 5 days in which observations were conducted plus 2 days). On day 8, males were removed and females were transferred to a fresh vial for an egg-laying period of 24 h, after which they were transferred to a new vial and allowed a second egg-laying period of 24 h. Note that females that died before or during egg laying, or that did not lay enough eggs to set up offspring experiments, were discarded (see below for details). Egg-laying periods were limited to 24 h to keep larval density low, thereby avoiding effects of overcrowding on the developing offspring (egg density during first 24-h egg-laying period, mean ± SE: 38.21 ± 1.32), and there were no significant differences in egg density between treatments (linear model with treatment as fixed effects: Treatment, *F*_1,47_ = 0.838, *P* = 0.3065).

Eggs from these two vials from each female were allowed to develop and, upon emergence, the adult offspring were allocated to one of three treatments: (a) focal daughter or son in isolation (two replicate sons and daughters per family); (b) focal son in a group with two *spa* males and one *spa* female (all three males were unrelated and unfamiliar to each other and to the female, two replicate sons per family; *spa* flies were collected as eggs from a *spa* population cage and reared at standard density; Clancy and Kennington 2001); and (c) focal daughter (two replicate daughters per family) placed with three males that were all unrelated and unfamiliar to each other and to the female (males collected as eggs from a population cage and reared at standard density; Clancy and Kennington 2001). Placing males with *spa* females and rival males meant that we could assign paternity using the eye phenotype of the subsequent offspring to the focal males (i.e., the sons of females from generation 1) relative to their *spa* rivals. We monitored offspring vials daily for mortality and transferred flies to fresh vials every 3 or 5–6 days (for vials containing four flies or a fly in isolation, respectively). Overall, a total of 8 offspring (four males and four females) per family were subjected to the above treatments. Sixteen ABC+ and 10 AAA+ parental vials were discarded from the transgenerational experiment (but not from behavioral observations of parental flies) due to male or female parental deaths during the days of interactions, so they did not contribute offspring to transgenerational assays. A total of 28 AAA+ and 29 ABC+ parental (generation 1) females produced enough offspring to set up all the treatments in generation 2.

To compare the early productivity of daughters from the ABC+ and AAA+ parental treatments, we allowed eggs laid by the daughters (treatment “a”, above) over the first 3 days (i.e., collected on day 4) to develop and counted their adult offspring. Similarly, to measure the reproductive success of sons from the ABC+ and AAA+ treatments, we allowed eggs laid in the first 3 days (i.e., collected on day 4; treatment “c” above) to develop and scored the resulting adult offspring eye phenotype as *spa* (rival male) or wild type (focal male).

### Statistical analysis

Differences in aggression, courtship rates, and courtship intensity were explored by fitting separate generalized linear mixed models (GLMMs) with treatment as a fixed factor and set (i.e., the group of three families used to produce ABC vs. AAA treatments, see above) as a random factor. Aggression event rates were slightly right-skewed, and hence, for this variable, the model was re-fit after square-root transforming data, which did not change the qualitative outcome of analyses.

To explore differences in survival and life span across treatments, we used a Cox’s proportional hazards mixed model including treatment as fixed effect and family (i.e., each family contributed two males and two females to each aging treatment) as a random effect. Differences in the reproductive success of male offspring (i.e., the proportion of offspring from focal wild type vs. competitor *spa* males) among treatments were analyzed fitting a quasibinomial GLM (due to over-dispersion of the binomial model, which also precluded an analysis using a GLMM with family as a random factor). Differences in early daughter offspring productivity were analyzed using a GLMM with family as a random variable.

Finally, to explore the potential relationship between parental behavior and daughter survival, we conducted a series of post hoc tests. First, we used GLMMs to explore the relationship between aggression rate and courtship rate and courtship intensity and their interaction with treatment (fixed effects), and family (random effect), with the objective of examining how these behavioral measures correlated within each vial, and across treatments. Second, we fitted a GLMM with daughter life span as the response variable, courtship rate, courtship intensity, aggression rate, and the interaction between aggression rate and courtship intensity (detected in the former analysis) as fixed factors and family as a random effect.

Heteroscedasticity assumptions were checked in all cases, and all error distributions were Gaussian unless specified otherwise. The potential influence of outliers was analyzed by re-fitting models on alpha-winsorized data (outliers had no undue influence), and all reported statistics are for raw data. All reported probabilities are two-tailed. Analyses were conducted in R v. 3.1.1 (R Core Team 2014. R: A language and environment for statistical computing. R Foundation for Statistical Computing, 2014).

## Results

### Behavioral assays in parental flies (generation 1)

**Figure 2:**
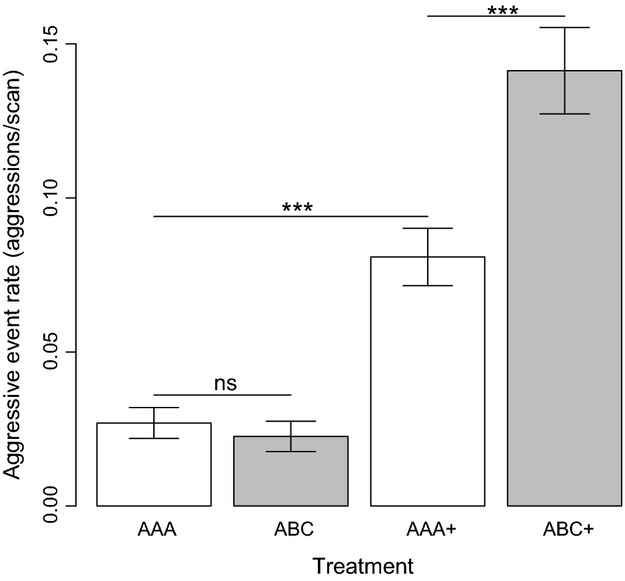
Differences in the frequency of male–male aggressive events among groups of related-familiar (AAA) or unrelated-unfamiliar male flies (ABC) in the presence (+) or the absence of a female in generation 1. Bars indicate means and lines indicate standard errors. ***, *P* < 0.001. ns, *P* > 0.05.

Treatment significantly affected the frequency of male–male aggression (df = 3, *χ*^2^ = 89.858, *P* < 0.001; Fig.2). A post hoc HSD Tukey test showed that male aggression rate was significantly higher among unrelated-unfamiliar males than among related-familiar males in the presence of a female (mean aggression rates ± SE, AAA+ 0.081 ± 0.009, ABC+ 0.141 ± 0.014, *z* = 4.764, *P* < 0.001, Cohen’s D = 0.73; estimated difference between ABC+ and AAA+ aggression rates: 0.060 ±0.013). Aggression was also significantly higher when related-familiar males were exposed to a female (AAA+) than when either related-familiar or unrelated-unfamiliar males were kept in the absence of a female (AAA, estimate ± SE, 0.054 ± 0.013, *z* = 4.225, *P* < 0.001; ABC 0.058 ± 0.013, *z* = 4.589, *P* < 0.001). Male–male aggression rates were low in the absence of females and did not differ between treatments (AAA vs. ABC treatments, estimate ± SE = −0.004 ± 0.013, *z* = −0.336, *P* = 0.987; Fig.2). To complement this finding, we performed a post hoc time-explicit analysis by fitting a GLMM with treatment, day and treatment × day interaction as fixed effects, and set as random factor. We found that male–male aggression tended to fluctuate over successive days, but increased steadily during the last days of observation in unrelated-unfamiliar males (ABC+), while remaining low among related-familiar males (AAA+; day × treatment df = 1, *χ*^2^ = 10.611, *P* = 0.001).

**Figure 3:**
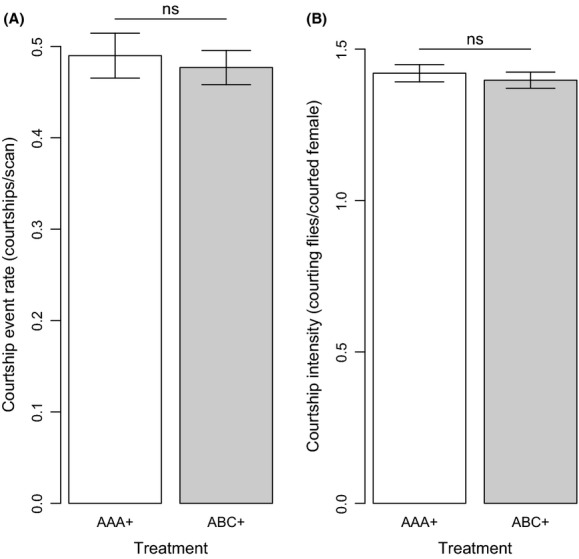
(A) The frequency (number of courtship events observed per scan) and (B) intensity (number of courting males per courted female) of male courtship in groups of related-familiar (AAA+) and unrelated-unfamiliar (ABC+) flies in generation 1. Bars indicate means, and lines indicate standard errors. ns, *P* > 0.05.

We did not detect an effect of male relatedness/familiarity on either courtship rate (df = 1, *χ*^2^ = 0.080, *P* = 0.777) or courtship intensity (df = 1, *χ*^2^ = 0.363, *P* = 0.547; Fig.3). However, courtship rate was positively and significantly associated with aggression rate across treatments (*χ*^2^ = 8.846, df = 1, *P* = 0.003). Courtship intensity was also positively associated with aggression rate, but via an interaction with treatment, such that this relationship was only significant in unrelated-unfamiliar vials (*χ*^2^ = 8.091, df = 1, *P* = 0.005; Appendix S3A and B).

### Transgenerational effects on offspring (generation 2)

**Figure 4:**
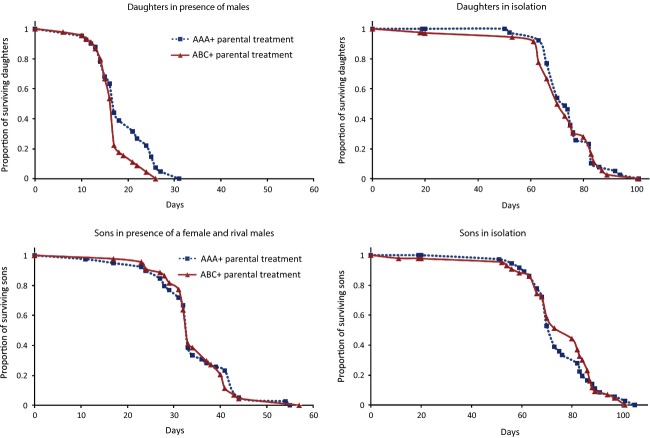
The survival of sons and daughters (generation 2) from mothers subject to AAA+ (blue lines) vs. ABC+ (red lines) parental treatments (generation 1), when housed with the opposite sex (focal daughter housed with three unrelated-unfamiliar males; focal son housed with two unrelated-unfamiliar rival males and one unrelated female) or in isolation.

When they were exposed to groups of unrelated-unfamiliar males, daughters of mothers that had also been exposed to unrelated-unfamiliar males (ABC+ treatment) died faster than daughters of mothers exposed to related-familiar males (AAA+, *χ*^2^ = 4.965, df = 1, *P* = 0.026; Fig.4). However, this difference between parental treatments (AAA+ vs. ABC+) was not apparent when daughters were kept in isolation (*χ*^2^ = 0.050, df = 1, *P* = 0.824; Fig.4).

We found no effect of parental courtship rate levels on daughter survival (*χ*^2^ = 0.598, df = 1, *P* = 0.440), but a marginal relationship between daughter survival and the interaction between aggression rate and courtship intensity (*χ*^2^ = 3.593, df = 1, *P* = 0.058). Exploration of this interaction suggests that daughters from generation 1 females that were subject to both high levels of courtship intensity and male–male aggression (which were correlated in unrelated-unfamiliar vials; Appendix S3A and B) suffered lower life spans (Appendix S3C and D).

We did not detect any parental treatment effect on the life span of sons, regardless of whether they were kept with two unrelated-unfamiliar males and a female (*χ*^2^ = 0.054, df = 1, *P* = 0.816; Fig.4) or in isolation (*χ*^2^ = 0.052, df = 1, *P* = 0.820; Fig.4). We did not find significant parental treatment differences in the relative mating success of sons when in competition against two unrelated-unfamiliar *spa* males (mean proportion of paternity ± SE, AAA+ = 0.67 ± 0.05, ABC+ = 0.71 ± 0.05; estimated ABC+ coefficient ± SE = 0.555 ± 0.523, *t* = 1.060, *P* = 0.293). Similarly, we did not find significant treatment differences in the early productivity of the daughters of AAA+ vs. ABC+ mothers (mean number of offspring produced in days 1–3 ± SE, AAA+ = 59.80 ± 2.50, ABC+ = 61.87 ± 2.09; *χ*^2^ = 0.317, *P* = 0.573).

## Discussion

In this study, we found that the degree of relatedness/familiarity among rival males modulates the intensity of male–male aggression, but only in the presence of females. Furthermore, we found that the effects of male relatedness and/or familiarity extend beyond a single generation: in the presence of males, the daughters of females that interacted with related, familiar males experienced longer life spans compared with the daughters of females that interacted with unrelated, unfamiliar males.

### Behavioral responses to the relatedness and familiarity of rivals

Our finding that groups of unrelated, unfamiliar males fight more than groups of familiar relatives is qualitatively similar to the findings of our previous study (Carazo et al. 2014). Quantitatively, the present study found that male relatedness/familiarity had a stronger effect—almost double—on male–male aggression than our previous study (see Appendix S1). This difference may be due to the addition of live yeast in the present study, which has previously been shown to increase male aggression/territoriality and female fecundity in *D. melanogaster* (Hoffmann and Cacoyianni 1990; Chippindale et al. 1993). The present study also shows that differences in male–male aggression are contingent on the presence of a female: in the absence of females, male aggression was low, and similar between groups of related-familiar and unrelated-unfamiliar males. This result is consistent with previous findings that male *D. melanogaster* adjust their aggressive and territorial behavior in response to immediate competition for mating opportunities (Hoffmann and Cacoyianni 1990). In contrast to our previous findings, we did not observe effects of male relatedness/familiarity on courtship activity, which could be due to differences in experimental conditions or due to limited power to detect the subtle differences in courtship behavior previously reported (see Appendix S1). Collectively, these results suggest that male *D. melanogaster* adjust their investment in intrasexual competition based on aspects associated with population structure (i.e., the relatedness and larval familiarity of male groups), but that these responses are also highly sensitive to socio-sexual and nutritional conditions.

The evolutionary significance of these behavioral responses remains unclear. Population structure can affect both local familiarity and local relatedness, frequently at the same time, creating a situation where developmental familiarity and relatedness are correlated in nature. The proximate cue used in this study to manipulate male perception of population structure was larval co-rearing, which may act as a proxy for both social familiarity and relatedness (see Hollis et al. 2015; Pizzari et al. 2015). Hence, in principle adaptive explanations of these behaviors encompass both direct fitness effects (i.e., those influencing a male’s offspring production) based on familiarity per se, and indirect fitness effects (i.e., those influencing a male’s inclusive fitness via the offspring production of relatives) based on relatedness. Both direct and indirect fitness effects may have contributed to the evolution and maintenance of the male behavioral plasticity reported here and in our previous study (Pizzari et al. 2015).

In theory, males might gain directly by reducing aggression toward socially familiar competitors irrespective of relatedness. Direct effects often evoked to explain cooperation among familiar nonkin include reciprocity, reputation, and communal defense against predators (e.g., Clutton-Brock 2009). It is, however, unclear whether these effects can play a role in *D. melanogaster* populations. Other mechanisms based on social familiarity, such as the “dear enemy” effect or the formation of dominance hierarchies, rely on habituation or learning, particularly during competitive interactions among adults. They are therefore unlikely to apply to this study in which adult familiarity was standardized across treatments. These effects also predict decreased aggression through time (with increasing social familiarity), which is not supported by our observations (Appendices S1 and S2).

Variation in female behavior might also modulate plastic male aggression via direct effects. For example, in the context of mate choice, “rare male” effects might make females more receptive in the presence of three unrelated (and hence more genetically dissimilar) males, which might in turn trigger more intense fighting and increased sexual harassment, leading to female harm. However, evidence for rare male effects in *Drosophila* is limited (Partridge 1988). On the contrary, several studies have indicated that *D. melanogaster* females may actually prefer to mate with related mates (Loyau et al. 2012; Robinson et al. 2012b), sexually familiar males or with socially novel males closely related with familiar males (Tan et al. 2013), which is inconsistent with this hypothesis. A further possibility based on direct effects is that plasticity in aggression arises as a by-product of a generalized “Coolidge effect,” whereby males may invest less in sexual competition if they perceive related rivals as weaker versions of themselves (Pizzari et al. 2015). This is not implausible, as male *D. melanogaster* have been shown to avoid mating with females previously mated by their own male relatives (Tan et al. 2013; Pizzari et al. 2015). However, the lack of consistent temporal patterns in aggression (see Appendices S1 and S2) is not consistent with this idea.

Males may also have evolved reduced aggression toward relatives as a result of indirect benefits. When local male competitors are more genetically related to each other than the population average and males can potentially increase local group productivity (and hence their own inclusive fitness) by reducing competitiveness, kin selection may favor reduced investment in competitive traits (Rankin 2011; Wild et al. 2011; Pizzari and Gardner 2012; Pizzari et al. 2015). This can happen, for example, because males are less likely to injure or impose other costs on their relatives or the mates of their relatives.

It is, however, unclear whether *D. melanogaster* populations display the level of population structure required to favor the evolution of such plasticity. It is likely that factors such as mixed paternity resulting from polyandry and larval dispersal will act to reduce local relatedness. This is especially relevant in laboratory-adapted populations such as ours, which have spent more than four decades (>1000 generations) in seemingly panmictic conditions. A possible explanation is that plasticity in response to the relatedness or familiarity of rivals is an ancestral trait, which evolved when natural populations were more viscous and which has been retained in some laboratory-adapted populations. In fact, laboratory-adapted *D. melanogaster* exhibit many plastic behavioral responses (e.g., in ejaculate allocation or mating duration) in socio-sexual contexts that are potentially functionally crucial in ancestral natural conditions, but unlikely to be of importance in the laboratory conditions in which they have been reared for decades (e.g., the presence or absence of a rival, of related vs. unrelated partners, or of sexually familiar vs. unfamiliar partners; Lize et al. 2013; Bretman et al. 2009, 2011; Tan et al. 2013). There is evidence that some natural populations of *D. melanogaster* display significant genetic structure (McInnis et al. 1982; Robinson et al. 2012a), suggesting that sensitivity to the relatedness and/or familiarity of rival males may have had adaptive value for male flies in their ancestral environment. In order to shed light into the evolutionary role of kin selection in modulating male–male competition and female harm in *Drosophila*, future work will need to characterize variation in plasticity with respect to social partner relatedness and familiarity across populations and relate this variation to differences in evolutionary history and population structure. Regardless of the mechanisms driving its evolution, the modulation of male aggression and female harm observed in *D. melanogaster* represents a proof of concept that population structure can have inclusive fitness implications broadly consistent with kin selection theory.

The proximate mechanisms that male flies use to discriminate between related-familiar and unrelated-unfamiliar rivals are likely to involve cues acquired through social familiarity. It has been recently shown that when reared apart from each other as larvae (and hence unfamiliar), related males can be just as harmful to females as unrelated males (Hollis et al. 2015), indicating that acquired cues associated with a shared larval environment are required for discrimination. Familiarity cues are widespread across taxa, including in insects (Wyatt 2014), and can modulate kin recognition whenever there is a statistical association between relatedness and familiarity in the wild (Sherman et al. 1997). We have previously shown that flies not only discriminate between sexually novel and sexually familiar partners, but also that they can also recognize socially novel individuals as related or unrelated to familiar partners, indicating that familiarity cues may be used to assess genetic relatedness (Tan et al. 2013). *Orco*^*1*^ mutant flies, which lack a co-receptor essential for olfaction, display reduced or no discrimination of familiar partners, novel individuals related to familiar partners, or flies from different laboratory strains, suggesting a role for olfactory cues (Billeter et al. 2012; Tan et al. 2013). However, we do not know whether familiarity cues are sufficient to generate these responses or whether other innate cues of genetic relatedness are also required. Recent experiments in three *Drosophila* species, including *D. melanogaster*, show that kin recognition is mediated by species-specific combinations of innate cues and cues acquired through a shared diet or rearing environment (Lize et al. 2013). It is crucial to note here that ultimate and proximate explanations based on direct and indirect effects are not mutually exclusive in structured populations, where viscosity generates a statistical association between familiarity and relatedness (Lehmann and Rousset 2010). In such cases, for example, kin biases are often based on familiarity-based proximate mechanisms (e.g., Penn and Frommen 2010). Future work is required to disentangle the proximate mechanisms regulating differential responses to related and familiar competitors in *D. melanogaster*. Specifically, studies are required that experimentally manipulate familiarity and relatedness independently in a fully balanced design.

### Transgenerational effects

The present study provides experimental evidence that male population structure (relatedness and familiarity) is associated with sex-specific transgenerational effects. In particular, we found increased life span in the daughters of females exposed to a group of related-familiar males, relative to daughters of females exposed to unrelated-unfamiliar males. Life span increased only when daughters themselves were exposed to males. This result does not appear to be due to trade-offs with early reproduction, because we detected no differences in early-life productivity between the groups. Furthermore, the fact that we did not detect differences in sons’ offspring output indicates that sons were not negatively affected in a way that would negate benefits to daughters. The fact that we did not detect any transgenerational effects in daughters kept in isolation suggests such effects are only evident when daughters are exposed to sexual harassment by groups of males. Thus, the transgenerational effect might specifically affect traits involved in resistance to male-induced harm (Holland and Rice 1999; Wigby and Chapman 2004).

Two nonmutually exclusive mechanisms could account for these transgenerational patterns. First, females exposed to unrelated males (ABC+) are likely to produce offspring that on average are less related to each other than the offspring of females exposed to full-sib brothers (AAA+). This difference in offspring relatedness may in turn exacerbate larval competition among offspring of ABC+ mothers (Ala-Honkola et al. 2011), predisposing them to vulnerability to stress in adult life. In this way, inclusive fitness arguments could explain the patterns observed in both parental and offspring generations.

Second, the observed transgenerational effects might be caused by parental effects operating through fathers or mothers (Gapp et al. 2014). Maternal effects via differential maternal investment might explain the patterns of transgenerational effects we observed. For example, female exposure to more intense male–male fighting and courtship intensity might be sufficient to impair maternal investment due to reduced female health. Consistent with this, Carazo et al. (2014) show that later in life, females exposed to unrelated-unfamiliar males tend to produce fewer viable offspring than females exposed to related-familiar males. Our current results suggest a link between daughter survival and the joint levels of male–male competition and courtship intensity experienced by mothers (Appendix S3C and D). Interestingly, male–male competition and courtship intensity were only significantly correlated in unrelated-unfamiliar groups (those exhibiting higher male–male competition), but not in related-familiar groups (Appendix S3A and B). This result fits nicely with the hypothesis that transgenerational costs to daughters were caused by a coordinated increase in the intensity of male–male competition and female harm in unrelated-unfamiliar groups (Carazo et al. 2014). Similarly, females can bear immunological costs from dealing with ejaculates from genetically dissimilar males in other species (Fedorka and Zuk 2005; Baer et al. 2006), and such costs might reduce the quantity or quality of maternal provisioning of eggs, with repercussions for offspring performance later in life. Likewise, there is substantial evidence that, in a variety of species, males can tailor their ejaculates according to perceived levels of competition (Edward et al. 2010; Perry et al. 2013), so paternal effects might have generated the transgenerational effects we observed via differential sperm or seminal protein investment in relation to the relatedness of rival males (see Priest et al. 2008b; Crean and Bonduriansky 2014). The nature and relative contribution of paternal and maternal effects to the transgenerational consequences of sexual interactions is an intriguing area for future study.

In conclusion, the results presented here and those reported in our recent study (Carazo et al. 2014) offer consistent evidence that *D. melanogaster* males respond plastically to male population structure, as reflected by local relatedness and/or familiarity, by modulating the intensity of male–male competition. This plasticity is associated with effects on females via both differences in female harm (Carazo et al. 2014; Hollis et al. 2015) and, as shown here, differences in the longevity of their daughters. Jointly, these results suggest that population structure can play an important yet previously unappreciated role in the evolution of sexual conflict by modulating male–male competition and female harm levels. Future studies should address the relative importance of direct (familiarity-based) vs. indirect (kin selection) implications in the evolution and maintenance of plasticity in sexual competitiveness in *D. melanogaster* and explore the relationships between population structure, male–male competition, and female harm levels across other taxa.

## Conflict of Interest

None declared.

## Supporting Information

**Appendix S1**. Daily frequency of male-male aggressive events among groups of related (AAA+) or unrelated male flies (ABC+) in the presence of a female. Bars indicate means and lines indicate standard errors.

**Appendix S2**. Daily frequency of male-male aggressive events in groups of males of increasing within-group relatedness in the presence of a female: three related males (AAA+), two related males and one unrelated male (AAB+) and three unrelated males (ABC+). Data from Carazo, P., Tan, C.K.W., Allen, F., Wigby, S. & Pizzari, T. 2014 Within-group male relatedness reduces harm to females in Drosophila. Nature, 505, 672-675. Bars indicate means and lines indicate standard errors.

**Appendix S3.** Within vial correlations between aggressive rate (frequency of scans where aggression was observed) and a) courtship intensity (average courting males when a female is courted) and b) courtship rate (frequency of scans where courtship was observed) across treatments. c-d) Interaction plots showing predicted values for daughter survival (in ABC+ groups) in relation to aggressive rate and courtship intensity, seen from opposite angles. Daughter survival increases with aggressive rate and courtship intensity when aggressive/courtship rate are low (d), and decreases with aggressive rate and courtship intensity when aggressive/courtship rates are high (c). Predicted values in c-d were calculated by fitting a GAM model using the vis.gam function in Simon Wood’s mgcv R package (Wood 2006).

**Appendix S4**. Complementary statistical tests (see Statistical analyses in Methods section of the main manuscript).
