# Supplementary figures and images for: Related male Drosophila melanogaster reared together as larvae fight less and sire longer lived daughters
Source: Ecol Evol. 2015 Jun 24;5(14):2787–97. doi: 10.1002/ece3.1549 (PMC4541986; doi:10.1002/ece3.1549)

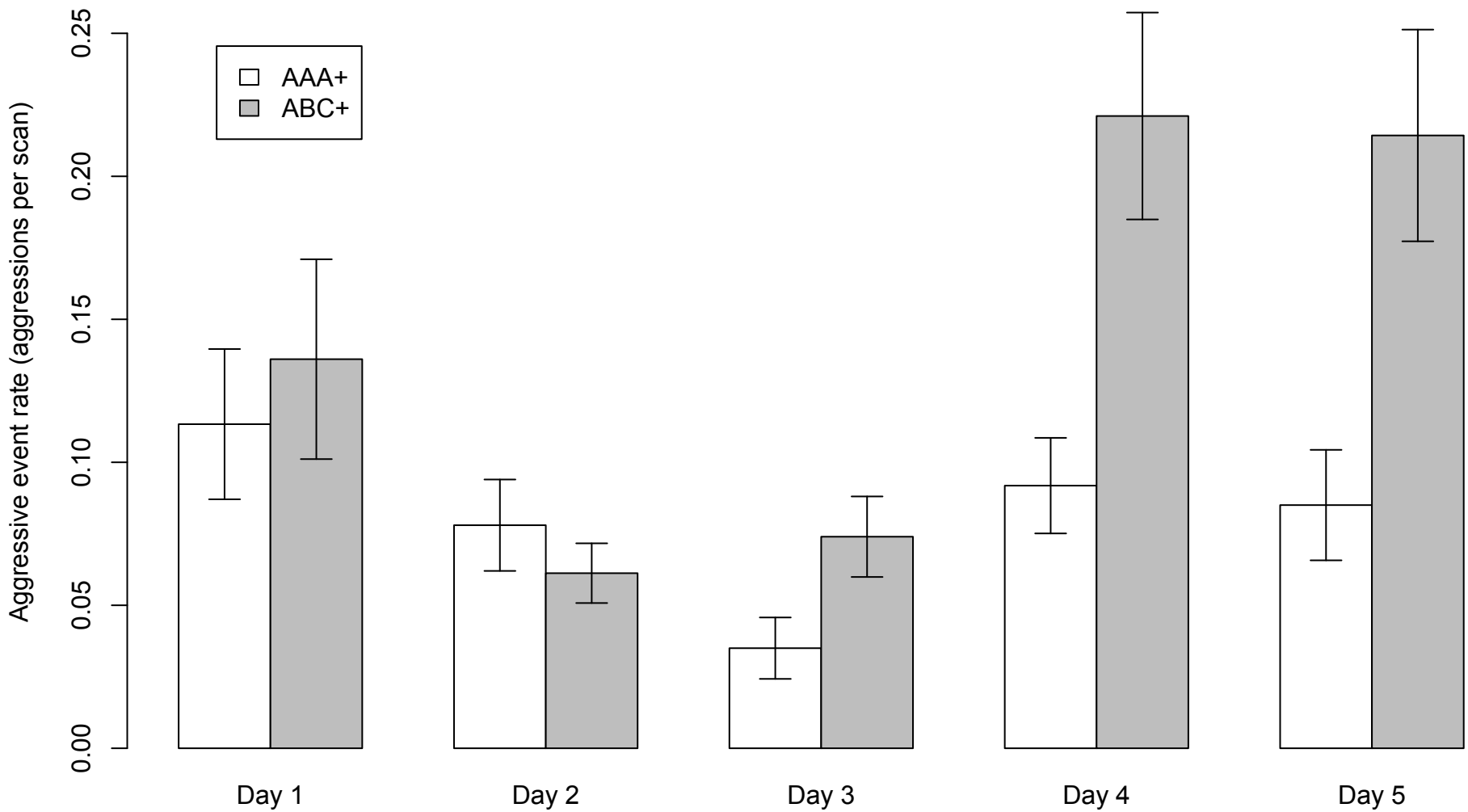

Supplement: Supplementary file 1 [file ece30005-2787-sd1.pdf]

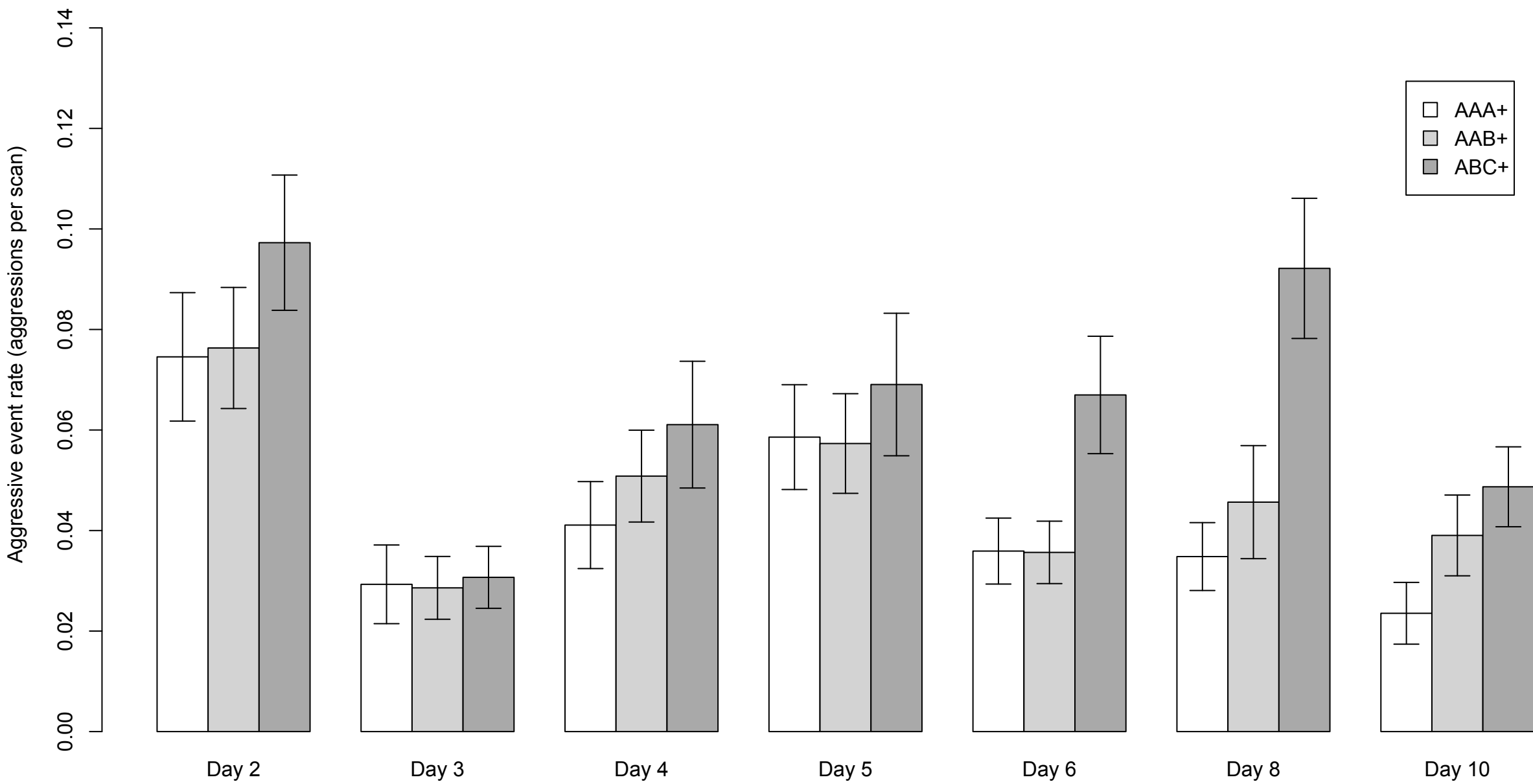

Supplement: Supplementary file 2 [file ece30005-2787-sd2.pdf]
