# Supplementary material for: Related male Drosophila melanogaster reared together as larvae fight less and sire longer lived daughters
Source: Ecol Evol. 2015 Jun 24;5(14):2787–97. doi: 10.1002/ece3.1549 (PMC4541986; doi:10.1002/ece3.1549)
Supplement: Supplementary file 4 [file ece30005-2787-sd4.docx]

**Appendix 1**. Daily frequency of male-male aggressive events among groups of related (AAA+) or unrelated male flies (ABC+) in the presence of a female. Bars indicate means and lines indicate standard errors.

**Appendix 2**. Daily frequency of male-male aggressive events in groups of males of increasing within-group relatedness in the presence of a female: three related males (AAA+), two related males and one unrelated male (AAB+) and three unrelated males (ABC+). Data from Carazo, P., Tan, C.K.W., Allen, F., Wigby, S. & Pizzari, T. 2014 Within-group male relatedness reduces harm to females in Drosophila. *Nature*, 505, 672-675. Bars indicate means and lines indicate standard errors.

**Appendix 3.** Within vial correlations between aggressive rate (frequency of scans where aggression was observed) and a) courtship intensity (average courting males when a female is courted) and b) courtship rate (frequency of scans where courtship was observed) across treatments. c-d) Interaction plots showing predicted values for daughter survival (in ABC+ groups) in relation to aggressive rate and courtship intensity, seen from opposite angles. Daughter survival increases with aggressive rate and courtship intensity when aggressive/courtship rate are low (d), and decreases with aggressive rate and courtship intensity when aggressive/courtship rates are high (c). Predicted values in c-d were calculated by fitting a GAM model using the vis.gam function in Simon Wood’s mgcv R package (Wood 2006).

**Appendix 4**. Complementary statistical tests (see Statistical analyses in Methods section of the main manuscript).

| **Variable** | **Test** | **Results (treatment effect)** |
| --- | --- | --- |
| Parental aggression event rate | GLMM on square-root transformed data | Df= 3, χ^2^ = 90.04, p < 0.001  Tukey:   - AAA+ vs. AAA, estimate ± SE = 0.13 ± 0.027, z = 5.003, p <0.001 - ABC vs. AAA, estimate ± SE = -0.013 ± 0.027, z = -0.496, p = 0.9601 - ABC+ vs. AAA, estimate ± SE = 0.23 ± 0.027, z = 8.646, p <0.001 - ABC vs. AAA+, estimate ± SE = -0.15 ± 0.026, z = -5.532, p <0.001 - ABC+ vs. AAA+, estimate ± SE = 0.098 ± 0.026, z = 3.709, p = 0.0012 - ABC+ vs. ABC, estimate ± SE = 0.244 ± 0.027, z = 9.193, p <0.001 |

**Achieved power to detect differences in courtship rate**: Assuming the same study design, the *post hoc* achieved power to detect differences in courtship intensity based on the effect size detected in our previous study [1] and the sample size of this study is low (0.53). Note that, due to practical limitations, in this study we reduced the number of observation days (from 10 to 5) and daily hours of observation (from 3 to 2h) in comparison with our previous study [1], which may have further reduced our power to detect differences in courtship behaviour.

**Quantitative differences in aggression rates between this and previous study** [1]: A direct comparison of the difference in aggression rates between related (AAA+) and unrelated males (ABC+) observed in the present and the previous study [1] revealed that the effect of treatment (i.e., the effects size of the difference in male-male aggression between AAA+ vs. ABC+) was almost twice as large in the present study:

- Current study: mean aggression rates ± SE, AAA+ 0.081 ± 0.009, ABC+ 0.141 ± 0.014, z = 4.764, p < 0.001, Cohen’s D = 0.73.
- Previous study: mean ± SE male-male aggression rates for the first five days from the previous study [1], AAA+, 0.047 ± 0.005, ABC+, 0.063 ± 0.007; Cohen’s D = 0.412.

Carazo, P., Tan, C.K.W., Allen, F., Wigby, S. & Pizzari, T. 2014 Within-group male relatedness reduces harm to females in Drosophila. *Nature* **505**, 672-675. (doi:10.1038/nature12949).

Wood, S.N. (2006) Generalized Additive Models: An Introduction with R. Chapman and Hall/CRC.
